# *N*′-[(1*E*)-4-Diethyl­amino-2-hy­droxy­benz­idene]benzohydrazide

**DOI:** 10.1107/S160053681103652X

**Published:** 2011-09-14

**Authors:** M. Prabhu, C. Meenakshi, G. Chakkaravarthi, G. Rajagopal

**Affiliations:** a Shasun Pharmaceuticals Ltd, Chennai 600 048, India; b Department of Chemistry, Government Arts College for Women (Autonomous), Madurai 625 002, India; c Department of Physics, CPCL Polytechnic College, Chennai 600 068, India; d Department of Chemistry, Government Arts College, Melur 625 106, India

## Abstract

In the title compound, C_18_H_21_N_3_O_2_, the dihedral angle between the phenyl and benzene rings is 36.85 (10)°. The methyl C atom of one of the ethyl groups is disordered over two positions with site occupancies of 0.810 (8) and 0.190 (8). The mol­ecular structure is stabilized by a classical intra­molecular O—H⋯N hydrogen bond. The crystal structure exhibits weak inter­molecular N—H⋯O, C—H⋯O and C—H⋯π inter­actions.

## Related literature

For the biological activity of Schiff base ligands, see: Kelley *et al.* (1995 ▶); Pandeya *et al.* (1999 ▶); Singh & Dash (1988 ▶); Tarafder *et al.* (2002 ▶). For related strucutures, see: Bahron *et al.* (2010 ▶); Manvizhi *et al.* (2010 ▶).
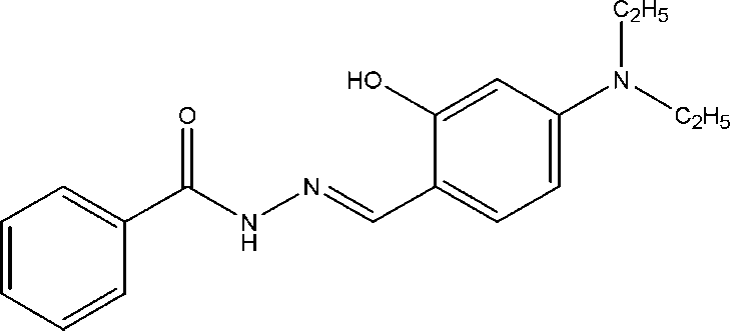


## Experimental

### 

#### Crystal data

C_18_H_21_N_3_O_2_
*M* *_r_* = 311.38
Monoclinic, 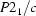

*a* = 10.591 (5) Å
*b* = 16.733 (6) Å
*c* = 9.671 (5) Å
β = 102.316 (5)°
*V* = 1674.4 (13) Å^3^
*Z* = 4
Mo *K*α radiation
μ = 0.08 mm^−1^
*T* = 295 K
0.28 × 0.24 × 0.20 mm

#### Data collection

Oxford Diffraction Xcalibur Eos diffractometer
Absorption correction: multi-scan (*CrysAlis PRO*; Oxford Diffraction, 2009 ▶) *T*_min_ = 0.977, *T*_max_ = 0.984
9666 measured reflections
3884 independent reflections
2466 reflections with *I* > 2σ(*I*)
*R*_int_ = 0.031

#### Refinement

*R*[*F* ^2^ > 2σ(*F* ^2^)] = 0.072
*wR*(*F* ^2^) = 0.203
*S* = 1.05
3884 reflections
222 parameters
6 restraints
H-atom parameters constrained
Δρ_max_ = 0.43 e Å^−3^
Δρ_min_ = −0.40 e Å^−3^

### 

Data collection: *CrysAlis CCD* (Oxford Diffraction, 2009 ▶); cell refinement: *CrysAlis RED* (Oxford Diffraction, 2009 ▶); data reduction: *CrysAlis RED*; program(s) used to solve structure: *SHELXS97* (Sheldrick, 2008 ▶); program(s) used to refine structure: *SHELXL97* (Sheldrick, 2008 ▶); molecular graphics: *PLATON* (Spek, 2009 ▶); software used to prepare material for publication: *SHELXL97*.

## Supplementary Material

Crystal structure: contains datablock(s) I, global. DOI: 10.1107/S160053681103652X/rk2298sup1.cif

Structure factors: contains datablock(s) I. DOI: 10.1107/S160053681103652X/rk2298Isup2.hkl

Supplementary material file. DOI: 10.1107/S160053681103652X/rk2298Isup3.cml

Additional supplementary materials:  crystallographic information; 3D view; checkCIF report

## Figures and Tables

**Table 1:** Hydrogen-bond geometry (Å, °) *Cg*2 is the centroid of the C9–C14 ring.

*D*—H⋯*A*	*D*—H	H⋯*A*	*D*⋯*A*	*D*—H⋯*A*
O2—H2*A*⋯N2	0.82	1.92	2.643 (3)	147
N1—H1⋯O1^i^	0.86	2.10	2.926 (3)	160
C8—H8⋯O1^i^	0.93	2.50	3.293 (3)	144
C3—H3⋯*Cg*2^ii^	0.93	2.97	3.468 (5)	115

Symmetry codes: (i) 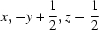; (ii) 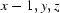.

**Fig. 1.**
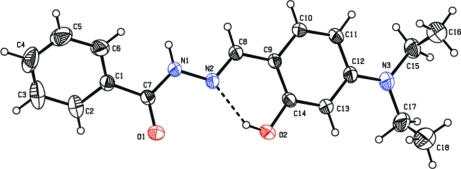
The molecular structure of title compound with the atom numbering scheme. Displacement ellipsoids are drawn at 30% probability level. For disordered ethyl group only major moiety is presented. H atoms are shown as a small spheres of arbitrary radius.

**Fig. 2.**
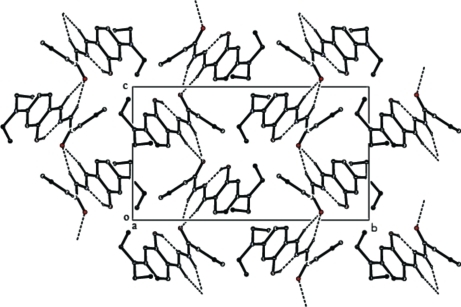
The crystal structure of title compound, viewed down a axis. Intermolecular hydrogen bonds are shown as dashed lines. H atoms not involved in hydrogen bonding have been omitted.

## supplementary crystallographic information

### Comment

In view of the biological actitivities of Schiff base ligands which are known to exhibit anti–viral, anti–cancer, anti–bacterial, anti–fungal, anti–inflammatory, anti–convulsant and anti–HIV activities (Pandeya *et al.*, 1999; Singh & Dash, 1988; Kelley *et al.*, 1995; Tarafder *et al.*, 2002), we report herein the molecular and crystal structures of the title compound.

The geometric parameters of the molecule of title compound (Fig.1) agree well with the reported similar structures (Bahron *et al.*, 2010; Manvizhi *et al.*, 2010). The dihedral angle between the phenyl ring (C1–C6) and the benzene ring (C9–C14) is 36.85 (10)°. The methyl C18 atom in the ethyl groups is disordered over two positions with site occupancies of 0.810 (8) and 0.190 (8).

The molecular structure is stabilized by weak intramolecular O2—H2A···N2 hydrogen bond and the crystal structure exhibit weak intermolecular N1—H1···O1^i^, C8—H8···O1^i^ and C3—H3···π (*Cg*2^ii^ is the centroid of C9–C14 ring) interactions (Fig. 2 & Table 1). Symmetry codes (i) and (ii) are indicated in Table 1.

### Experimental

The benzoic acid hydrazide (5 mmol) in methanol (10 ml) was stirred in a round bottom flask followed by drop wise addition of methanolic solution of 4–(diethylamino)salicylaldehyde (5 mmol). The reaction mixture was then refluxed for three hours and upon cooling to 273 K. A pale yellow crystalline solid precipitates from the mixture was separated out. Crystalline product was washed with ice cold ethanol and dried *in vacuo* over anhydrous CaCl_2_. Single crystals suitable for the *X*–ray diffraction were obtained by slow evaporation of a solution of the title compound in *DMF* at room temperature. Melting point 500 K.

### Refinement

The site occupancy factors for disordered C atom were refined as C18/C18A = 0.810 (8)/0.190 (8). H atoms were positioned geometrically with C—H = 0.93–0.97 Å, O—H = 0.82Å and N—H = 0.86Å and allowed to ride on their parent atoms, with *U*_iso_(H) = 1.5*U*_eq_(O), *U*_iso_(H) = 1.2*U*_eq_(N), *U*_iso_(H) = 1.5*U*_eq_(C) for methyl droups and *U*_iso_(H) = 1.2*U*_eq_(C) for other.

### Crystal data

**Table:**

C_18_H_21_N_3_O_2_	*F*(000) = 664
*M**_r_* = 311.38	*D*_x_ = 1.235 Mg m^−^^3^
Monoclinic, *P*2_1_/*c*	Melting point: 500 K
Hall symbol: -P 2ybc	Mo *K*α radiation, λ = 0.71073 Å
*a* = 10.591 (5) Å	Cell parameters from 4483 reflections
*b* = 16.733 (6) Å	θ = 2.9–29.1°
*c* = 9.671 (5) Å	µ = 0.08 mm^−^^1^
β = 102.316 (5)°	*T* = 295 K
*V* = 1674.4 (13) Å^3^	Block, pale yellow
*Z* = 4	0.28 × 0.24 × 0.20 mm

### Data collection

**Table:**

Oxford Diffraction Xcalibur Eos diffractometer	3884 independent reflections
Radiation source: fine–focus sealed tube	2466 reflections with *I* > 2σ(*I*)
graphite	*R*_int_ = 0.031
φ– and ω–scans	θ_max_ = 29.2°, θ_min_ = 2.9°
Absorption correction: multi-scan (*CrysAlis PRO*; Oxford Diffraction, 2009)	*h* = −14→13
*T*_min_ = 0.977, *T*_max_ = 0.984	*k* = −22→18
9666 measured reflections	*l* = −12→13

### Refinement

**Table:**

Refinement on *F*^2^	Secondary atom site location: difference Fourier map
Least-squares matrix: full	Hydrogen site location: inferred from neighbouring sites
*R*[*F*^2^ > 2σ(*F*^2^)] = 0.072	H-atom parameters constrained
*wR*(*F*^2^) = 0.203	*w* = 1/[σ^2^(*F*_o_^2^) + (0.0782*P*)^2^ + 0.8512*P*] where *P* = (*F*_o_^2^ + 2*F*_c_^2^)/3
*S* = 1.05	(Δ/σ)_max_ < 0.001
3884 reflections	Δρ_max_ = 0.43 e Å^−^^3^
222 parameters	Δρ_min_ = −0.40 e Å^−^^3^
6 restraints	Extinction correction: *SHELXL*, Fc^*^=kFc[1+0.001xFc^2^λ^3^/sin(2θ)]^-1/4^
Primary atom site location: structure-invariant direct methods	Extinction coefficient: 0.022 (3)

### Special details

**Table:**

Geometry. All s.u.'s (except the e.s.d. in the dihedral angle between two l.s. planes) are estimated using the full covariance matrix. The cell s.u.'s are taken into account individually in the estimation of s.u.'s in distances, angles and torsion angles; correlations between s.u.'s in cell parameters are only used when they are defined by crystal symmetry. An approximate (isotropic) treatment of cell s.u.'s is used for estimating s.u.'s involving l.s. planes.
Refinement. Refinement of *F*^2^ against ALL reflections. The weighted *R*–factor *wR* and goodness of fit *S* are based on *F*^2^, conventional *R*–factors *R* are based on *F*, with *F* set to zero for negative *F*^2^. The threshold expression of *F*^2^ > 2σ(*F*^2^) is used only for calculating *R*–factors(gt) *etc*. and is not relevant to the choice of reflections for refinement. *R*–factors based on *F*^2^ are statistically about twice as large as those based on *F*, and *R*–factors based on ALL data will be even larger.

### Fractional atomic coordinates and isotropic or equivalent isotropic displacement parameters (Å^2^)

**Table:**

	*x*	*y*	*z*	*U*_iso_*/*U*_eq_	Occ. (<1)
C1	0.3790 (2)	0.27667 (16)	0.7824 (2)	0.0476 (6)	
C2	0.2753 (3)	0.2435 (2)	0.8285 (3)	0.0669 (8)	
H2	0.2877	0.1980	0.8850	0.080*	
C3	0.1545 (3)	0.2771 (3)	0.7914 (5)	0.0950 (13)	
H3	0.0849	0.2538	0.8207	0.114*	
C4	0.1374 (4)	0.3448 (4)	0.7114 (4)	0.1128 (17)	
H4	0.0554	0.3672	0.6850	0.135*	
C5	0.2401 (5)	0.3807 (3)	0.6692 (4)	0.1231 (19)	
H5	0.2282	0.4282	0.6182	0.148*	
C6	0.3614 (3)	0.3455 (2)	0.7031 (3)	0.0831 (11)	
H6	0.4305	0.3684	0.6722	0.100*	
C7	0.5058 (2)	0.23618 (14)	0.8256 (2)	0.0416 (5)	
C8	0.7610 (2)	0.19544 (16)	0.6584 (2)	0.0477 (6)	
H8	0.7314	0.2260	0.5776	0.057*	
C9	0.8774 (2)	0.14947 (15)	0.6699 (2)	0.0436 (6)	
C10	0.9440 (2)	0.15018 (17)	0.5596 (2)	0.0510 (6)	
H10	0.9133	0.1823	0.4812	0.061*	
C11	1.0526 (2)	0.10543 (18)	0.5627 (3)	0.0559 (7)	
H11	1.0945	0.1082	0.4875	0.067*	
C12	1.1017 (2)	0.05524 (17)	0.6782 (3)	0.0524 (6)	
C13	1.0364 (2)	0.05409 (16)	0.7911 (3)	0.0512 (6)	
H13	1.0676	0.0222	0.8697	0.061*	
C14	0.9275 (2)	0.09940 (15)	0.7862 (2)	0.0449 (6)	
C15	1.2777 (3)	0.0098 (2)	0.5648 (3)	0.0690 (8)	
H15A	1.2157	0.0150	0.4757	0.083*	
H15B	1.3226	−0.0406	0.5630	0.083*	
C16	1.3733 (3)	0.0763 (2)	0.5773 (4)	0.0863 (10)	
H16A	1.3298	0.1264	0.5793	0.129*	
H16B	1.4140	0.0751	0.4976	0.129*	
H16C	1.4377	0.0700	0.6630	0.129*	
C17	1.2575 (4)	−0.0495 (4)	0.7989 (4)	0.1165 (17)	
H17A	1.1854	−0.0671	0.8385	0.140*	0.810 (8)
H17B	1.2922	−0.0961	0.7605	0.140*	0.810 (8)
H17C	1.3506	−0.0446	0.8124	0.140*	0.190 (8)
H17D	1.2359	−0.0233	0.8803	0.140*	0.190 (8)
N1	0.58174 (18)	0.23775 (13)	0.73010 (19)	0.0478 (5)	
H1	0.5590	0.2647	0.6531	0.057*	
N2	0.69687 (17)	0.19532 (13)	0.7574 (2)	0.0473 (5)	
N3	1.2080 (2)	0.00784 (18)	0.6801 (3)	0.0766 (8)	
O1	0.53870 (16)	0.20183 (12)	0.93992 (16)	0.0574 (5)	
O2	0.86897 (17)	0.09411 (12)	0.89825 (18)	0.0628 (6)	
H2A	0.8054	0.1234	0.8849	0.094*	
C18	1.3519 (5)	−0.0184 (4)	0.9069 (6)	0.121 (2)	0.810 (8)
H18A	1.3185	0.0276	0.9465	0.182*	0.810 (8)
H18B	1.4256	−0.0031	0.8699	0.182*	0.810 (8)
H18C	1.3772	−0.0581	0.9791	0.182*	0.810 (8)
C18A	1.241 (2)	−0.1276 (11)	0.827 (2)	0.122 (7)	0.190 (8)
H18D	1.2950	−0.1413	0.9165	0.183*	0.190 (8)
H18E	1.2651	−0.1598	0.7539	0.183*	0.190 (8)
H18F	1.1524	−0.1373	0.8287	0.183*	0.190 (8)

### Atomic displacement parameters (Å^2^)

**Table:**

	*U*^11^	*U*^22^	*U*^33^	*U*^12^	*U*^13^	*U*^23^
C1	0.0438 (13)	0.0650 (16)	0.0350 (10)	0.0059 (11)	0.0108 (9)	−0.0109 (11)
C2	0.0472 (15)	0.072 (2)	0.085 (2)	−0.0064 (14)	0.0217 (14)	−0.0232 (16)
C3	0.0463 (17)	0.121 (3)	0.119 (3)	−0.001 (2)	0.0213 (19)	−0.053 (3)
C4	0.071 (2)	0.185 (5)	0.080 (2)	0.063 (3)	0.0093 (19)	−0.023 (3)
C5	0.127 (3)	0.170 (4)	0.083 (2)	0.097 (3)	0.047 (2)	0.046 (3)
C6	0.086 (2)	0.106 (3)	0.0680 (18)	0.044 (2)	0.0400 (17)	0.0290 (18)
C7	0.0392 (11)	0.0510 (14)	0.0347 (10)	−0.0056 (10)	0.0080 (9)	−0.0076 (10)
C8	0.0398 (12)	0.0637 (16)	0.0396 (11)	0.0020 (11)	0.0084 (9)	0.0022 (11)
C9	0.0342 (11)	0.0578 (15)	0.0393 (11)	−0.0021 (10)	0.0088 (9)	0.0000 (10)
C10	0.0419 (12)	0.0728 (18)	0.0390 (11)	0.0034 (12)	0.0102 (10)	0.0084 (11)
C11	0.0470 (14)	0.0810 (19)	0.0441 (13)	0.0054 (13)	0.0199 (10)	0.0039 (12)
C12	0.0401 (13)	0.0704 (18)	0.0479 (13)	0.0053 (12)	0.0122 (10)	0.0003 (12)
C13	0.0427 (12)	0.0659 (17)	0.0462 (12)	0.0059 (12)	0.0122 (10)	0.0089 (12)
C14	0.0386 (12)	0.0583 (15)	0.0398 (11)	−0.0051 (11)	0.0129 (9)	0.0006 (10)
C15	0.0591 (16)	0.081 (2)	0.0721 (18)	0.0123 (16)	0.0255 (14)	−0.0058 (16)
C16	0.079 (2)	0.093 (3)	0.089 (2)	−0.003 (2)	0.0219 (18)	0.006 (2)
C17	0.080 (2)	0.200 (5)	0.078 (2)	0.077 (3)	0.0359 (17)	0.044 (2)
N1	0.0394 (10)	0.0662 (14)	0.0391 (9)	0.0089 (9)	0.0115 (8)	0.0044 (9)
N2	0.0364 (10)	0.0636 (14)	0.0422 (10)	0.0034 (9)	0.0094 (8)	−0.0003 (9)
N3	0.0568 (14)	0.110 (2)	0.0699 (15)	0.0288 (14)	0.0290 (12)	0.0117 (13)
O1	0.0563 (10)	0.0785 (13)	0.0387 (9)	0.0050 (9)	0.0128 (7)	0.0050 (8)
O2	0.0574 (11)	0.0875 (14)	0.0505 (10)	0.0146 (10)	0.0272 (8)	0.0179 (9)
C18	0.108 (4)	0.140 (5)	0.121 (4)	0.037 (3)	0.036 (2)	0.043 (3)
C18A	0.110 (17)	0.195 (10)	0.058 (11)	0.065 (16)	0.010 (10)	0.029 (14)

### Geometric parameters (Å, °)

**Table:**

C1—C6	1.374 (4)	C14—O2	1.362 (3)
C1—C2	1.387 (4)	C15—N3	1.464 (3)
C1—C7	1.483 (3)	C15—C16	1.492 (5)
C2—C3	1.373 (5)	C15—H15A	0.9700
C2—H2	0.9300	C15—H15B	0.9700
C3—C4	1.363 (6)	C16—H16A	0.9600
C3—H3	0.9300	C16—H16B	0.9600
C4—C5	1.378 (7)	C16—H16C	0.9600
C4—H4	0.9300	C17—C18A	1.353 (16)
C5—C6	1.387 (5)	C17—C18	1.384 (7)
C5—H5	0.9300	C17—N3	1.502 (5)
C6—H6	0.9300	C17—H17A	0.9700
C7—O1	1.229 (3)	C17—H17B	0.9700
C7—N1	1.348 (3)	C17—H17C	0.9700
C8—N2	1.287 (3)	C17—H17D	0.9700
C8—C9	1.437 (3)	N1—N2	1.387 (3)
C8—H8	0.9300	N1—H1	0.8600
C9—C10	1.399 (3)	O2—H2A	0.8200
C9—C14	1.412 (3)	C18—H17C	1.0114
C10—C11	1.367 (3)	C18—H17D	1.2036
C10—H10	0.9300	C18—H18A	0.9600
C11—C12	1.406 (4)	C18—H18B	0.9600
C11—H11	0.9300	C18—H18C	0.9600
C12—N3	1.374 (3)	C18A—H18D	0.9600
C12—C13	1.411 (3)	C18A—H18E	0.9600
C13—C14	1.372 (3)	C18A—H18F	0.9600
C13—H13	0.9300		
			
C6—C1—C2	119.5 (3)	H16A—C16—H16B	109.5
C6—C1—C7	123.2 (2)	C15—C16—H16C	109.5
C2—C1—C7	117.3 (2)	H16A—C16—H16C	109.5
C3—C2—C1	120.7 (4)	H16B—C16—H16C	109.5
C3—C2—H2	119.7	C18A—C17—C18	108.4 (9)
C1—C2—H2	119.7	C18A—C17—N3	136.9 (10)
C4—C3—C2	119.5 (4)	C18—C17—N3	114.5 (5)
C4—C3—H3	120.3	C18A—C17—H17A	59.0
C2—C3—H3	120.3	C18—C17—H17A	108.6
C3—C4—C5	120.9 (3)	N3—C17—H17A	108.6
C3—C4—H4	119.6	C18A—C17—H17B	51.1
C5—C4—H4	119.6	C18—C17—H17B	108.6
C4—C5—C6	119.6 (4)	N3—C17—H17B	108.6
C4—C5—H5	120.2	H17A—C17—H17B	107.6
C6—C5—H5	120.2	C18A—C17—H17C	102.8
C1—C6—C5	119.8 (3)	C18—C17—H17C	46.9
C1—C6—H6	120.1	N3—C17—H17C	103.2
C5—C6—H6	120.1	H17A—C17—H17C	146.7
O1—C7—N1	122.0 (2)	H17B—C17—H17C	70.2
O1—C7—C1	122.2 (2)	C18A—C17—H17D	102.4
N1—C7—C1	115.8 (2)	C18—C17—H17D	58.4
N2—C8—C9	121.5 (2)	N3—C17—H17D	103.2
N2—C8—H8	119.3	H17A—C17—H17D	58.3
C9—C8—H8	119.3	H17B—C17—H17D	148.1
C10—C9—C14	116.5 (2)	H17C—C17—H17D	105.2
C10—C9—C8	120.0 (2)	C7—N1—N2	119.22 (19)
C14—C9—C8	123.4 (2)	C7—N1—H1	120.4
C11—C10—C9	122.5 (2)	N2—N1—H1	120.4
C11—C10—H10	118.7	C8—N2—N1	116.09 (19)
C9—C10—H10	118.7	C12—N3—C15	121.5 (2)
C10—C11—C12	120.7 (2)	C12—N3—C17	122.0 (2)
C10—C11—H11	119.6	C15—N3—C17	116.5 (2)
C12—C11—H11	119.6	C14—O2—H2A	109.5
N3—C12—C11	121.2 (2)	H17C—C18—H17D	87.7
N3—C12—C13	121.1 (2)	C17—C18—H18A	109.5
C11—C12—C13	117.6 (2)	H17C—C18—H18A	140.6
C14—C13—C12	120.8 (2)	H17D—C18—H18A	72.1
C14—C13—H13	119.6	C17—C18—H18B	109.5
C12—C13—H13	119.6	H17C—C18—H18B	68.6
O2—C14—C13	117.3 (2)	H17D—C18—H18B	144.4
O2—C14—C9	121.0 (2)	C17—C18—H18C	109.5
C13—C14—C9	121.7 (2)	H17C—C18—H18C	107.8
N3—C15—C16	113.5 (3)	H17D—C18—H18C	102.8
N3—C15—H15A	108.9	C17—C18A—H18D	109.5
C16—C15—H15A	108.9	C17—C18A—H18E	109.5
N3—C15—H15B	108.9	H18D—C18A—H18E	109.5
C16—C15—H15B	108.9	C17—C18A—H18F	109.5
H15A—C15—H15B	107.7	H18D—C18A—H18F	109.5
C15—C16—H16A	109.5	H18E—C18A—H18F	109.5
C15—C16—H16B	109.5		
			
C6—C1—C2—C3	2.3 (4)	C12—C13—C14—O2	179.0 (2)
C7—C1—C2—C3	−179.3 (3)	C12—C13—C14—C9	−0.8 (4)
C1—C2—C3—C4	−1.6 (5)	C10—C9—C14—O2	−179.5 (2)
C2—C3—C4—C5	−1.0 (6)	C8—C9—C14—O2	−2.3 (4)
C3—C4—C5—C6	2.9 (7)	C10—C9—C14—C13	0.3 (4)
C2—C1—C6—C5	−0.4 (5)	C8—C9—C14—C13	177.5 (2)
C7—C1—C6—C5	−178.7 (3)	O1—C7—N1—N2	4.1 (3)
C4—C5—C6—C1	−2.1 (6)	C1—C7—N1—N2	−174.4 (2)
C6—C1—C7—O1	145.2 (3)	C9—C8—N2—N1	−175.9 (2)
C2—C1—C7—O1	−33.2 (3)	C7—N1—N2—C8	176.0 (2)
C6—C1—C7—N1	−36.4 (3)	C11—C12—N3—C15	2.4 (4)
C2—C1—C7—N1	145.3 (2)	C13—C12—N3—C15	−178.8 (3)
N2—C8—C9—C10	179.3 (2)	C11—C12—N3—C17	−176.6 (4)
N2—C8—C9—C14	2.1 (4)	C13—C12—N3—C17	2.2 (5)
C14—C9—C10—C11	−0.3 (4)	C16—C15—N3—C12	82.8 (4)
C8—C9—C10—C11	−177.6 (3)	C16—C15—N3—C17	−98.1 (4)
C9—C10—C11—C12	0.8 (4)	C18A—C17—N3—C12	93.6 (15)
C10—C11—C12—N3	177.6 (3)	C18—C17—N3—C12	−92.3 (4)
C10—C11—C12—C13	−1.2 (4)	C18A—C17—N3—C15	−85.5 (15)
N3—C12—C13—C14	−177.6 (3)	C18—C17—N3—C15	88.6 (4)
C11—C12—C13—C14	1.2 (4)		

### Hydrogen-bond geometry (Å, °)

**Table:**

Cg2 is the centroid of the C9–C14 ring.

**Table:**

*D*—H···*A*	*D*—H	H···*A*	*D*···*A*	*D*—H···*A*
O2—H2A···N2	0.82	1.92	2.643 (3)	147
N1—H1···O1^i^	0.86	2.10	2.926 (3)	160
C8—H8···O1^i^	0.93	2.50	3.293 (3)	144
C3—H3···Cg2^ii^	0.93	2.97	3.468 (5)	115

Symmetry codes: (i) *x*, −*y*+1/2, *z*−1/2; (ii) *x*−1, *y*, *z*.
